# Development and assessment of the Alberta Context Tool

**DOI:** 10.1186/1472-6963-9-234

**Published:** 2009-12-15

**Authors:** Carole A Estabrooks, Janet E Squires, Greta G Cummings, Judy M Birdsell, Peter G Norton

**Affiliations:** 1 Faculty of Nursing, University of Alberta, Edmonton, Alberta, Canada; 2 On Management Health Group, Calgary, Alberta Canada; 3 Department of Family Medicine, University of Calgary, Calgary, Alberta, Canada

## Abstract

**Background:**

The context of healthcare organizations such as hospitals is increasingly accepted as having the potential to influence the use of new knowledge. However, the mechanisms by which the organizational context influences evidence-based practices are not well understood. Current measures of organizational context lack a theory-informed approach, lack construct clarity and generally have modest psychometric properties. This paper presents the development and initial psychometric validation of the Alberta Context Tool (ACT), an eight dimension measure of organizational context for healthcare settings.

**Methods:**

Three principles guided the development of the ACT: substantive theory, brevity, and modifiability. The Promoting Action on Research Implementation in Health Services (PARiHS) framework and related literature were used to guide selection of items in the ACT. The ACT was required to be brief enough to be tolerated in busy and resource stretched work settings and to assess concepts of organizational context that were potentially *modifiable*. The English version of the ACT was completed by 764 nurses (752 valid responses) working in seven Canadian pediatric care hospitals as part of its initial validation. Cronbach's alpha, exploratory factor analysis, analysis of variance, and tests of association were used to assess instrument reliability and validity.

**Results:**

Factor analysis indicated a 13-factor solution (accounting for 59.26% of the variance in 'organizational context'). The composition of the factors was similar to those originally conceptualized. Cronbach's alpha for the 13 factors ranged from .54 to .91 with 4 factors performing below the commonly accepted alpha cut off of .70. Bivariate associations between instrumental research utilization levels (which the ACT was developed to predict) and the ACT's 13 factors were statistically significant at the 5% level for 12 of the 13 factors. Each factor also showed a trend of increasing mean score ranging from the lowest level to the highest level of instrumental research use, indicating construct validity.

**Conclusions:**

To date, no completely satisfactory measures of organizational context are available for use in healthcare. The ACT assesses several core domains to provide a comprehensive account of organizational context in healthcare settings. The tool's strengths are its brevity (allowing it to be completed in busy healthcare settings) and its focus on dimensions of organizational context that are modifiable. Refinements of the instrument for acute, long term care, and home care settings are ongoing.

## Background

Organizational context can be defined as "...the environment or setting in which people receive healthcare services, or in the context of getting research evidence into practice, the environment or setting in which the proposed change is to be implemented" [[1], p. 299]. Organizational context is widely considered to be an important influence on the successful implementation of research evidence in healthcare settings [1-4]. However, relatively little empirical evidence exists to support this claim. Further, its measurement has not been adequately addressed. In this paper, we report the first major assessment of a newly developed instrument, the Alberta Context Tool (ACT), designed to parsimoniously measure organizational context as perceived by healthcare providers working in complex healthcare settings.

The Alberta Context Tool (ACT) was developed with a specific purpose in mind and this shaped the approach taken to expanding our understanding of the construct of 'context'. Given our belief that organizational context is a central influence on the effective use of clinically relevant research evidence by healthcare providers, we sought to develop a tool that would allow us to assess context validly and reliably within complex healthcare settings where care is provided to patients. The resulting context measure was intended for administration at the level of the individual healthcare provider to determine their perception of context as it applies to a patient care unit or organization (e.g., hospital), depending on the individual's context of care delivery.

In the development of the ACT we tried to balance, to the extent possible, three principles: a substantive theory, brevity, and modifiability. We used the *Promoting Action on Research Implementation in Health Services *(PARiHS) framework to conceptualize organizational context. When the framework did not provide direction, we operationalized concepts from related literature (e.g., [5-8]). The PARiHS framework provides a broad conceptualization of how research implementation occurs in organizational settings. In the index paper for PARiHS [9] successful research implementation was proposed to result from the interplay and interdependence of three core elements: (1) evidence, (2) facilitation, and (3) context. We were interested in the context domain.

Context, in the PARiHS framework, is construed generally as the work setting and more specifically embodies three domains: culture, leadership and evaluation. *Culture *is defined as "the forces at work, which give the physical environment a character and feel" [9,10]. Subsequent exploration into the concept of 'culture' by McCormack and colleagues [11] resulted in further refinement of the definition of culture to encompass the prevailing beliefs and values, as well as consistency in these values and a receptivity to change, among members of an organizational setting.

The PARiHS framework defines *leadership *as the "nature of human relationships" [[11], p.98] with effective leadership giving rise to clear roles, effective teamwork and organizational structures, and involvement by organizational members in decision making and learning. This closely resembles 'transformational leadership'[11], a broad term reflecting leaders thought to be among the most effective leaders because they are able to transpose their ideas and beliefs into collective beliefs which eventually become assumptions and part of a unit's culture [11-13]. Emotionally intelligent leadership styles are one form of leadership consistent with transformational styles because they focus on how leaders manage their own emotions and their relationships with others both individually and in larger social settings [14].

*Evaluation *is described in the PARiHS framework as feedback mechanisms (individual and system level), sources, and methods for evaluation [9]. Audit (data gathered about the processes and/or outcomes of patient care) coupled with feedback (data provided to members of the organization) is one of the most commonly applied evaluation methods in healthcare organizations. Research implementation is hypothesized by the PARiHS developers to be most successful when evaluation occurs routinely.

A recent paper by the PARiHS group suggests that a fourth contextual component, *resources*, is important to the implementation of research findings. In 2004, Rycroft-Malone and colleagues [15] interviewed staff nurses, nurse managers, and other implementation 'experts' at two acute care agencies in the United Kingdom and identified time, equipment, and clinical skills as resources needed to implement research findings. They also identified the complexity of relationships among these resources.

## Methods

### Development of the ACT

In developing the ACT we worked to make it brief enough to be tolerated in busy and resource stretched work settings. This decision made ACT development of necessity pragmatic. We also chose to focus on concepts of organizational context that were potentially *modifiable*. Therefore we did not include concepts that could not be a focus of future research implementation intervention studies. Development of the ACT occurred in four phases: (1) selection of the conceptual framework, (2) conceptual refinement, (3) item construction, and (4) feasibility assessment. Time to complete the ACT was assessed as part of the feasibility assessment; the ACT was administered to five nurses with a documented mean completion time of 20.7 minutes. Additional detail on the development of the ACT is described elsewhere [16]. Following feasibility assessment, an index version (76 items) of the ACT covering eight dimensions of organizational context was developed. The initial (index) ACT tool was then pre-tested with 453 healthcare professionals (152 nurses, 36 physicians, 181 allied health professionals, 46 educators and specialists, 38 managers) in four acute care teaching hospitals in Alberta, Canada. Based on this pre-test, the instrument was revised and reduced from 76 to 56 items. Details of the ACT refinement can be found in Additional File 1. The refined ACT consists of 56 items reflecting the following eight contextual dimensions: culture (6 items), leadership (6 items), evaluation (6 items), social capital (6 items), informal interactions (7 items), formal interactions (5 items), structural and electronic resources (11 items), and organizational slack (9 items representing three sub-concepts - time, space, human resources). Definitions of the eight context dimensions, along with our hypotheses about their association with research implementation (i.e., research utilization) are listed in Table 1.

**Table 1:** Concepts in the ACT survey

Concept	Definition	Hypothesis	Sample item
Leadership ^1^	The actions of formal leaders in an organization (unit) to influence change and excellence in practice, items generally reflect emotionally intelligent leadership	**H1**: Care providers who perceive more positive (emotionally intelligent) unit leadership report higher research use	Calmly handles stressful situations
Culture ^1^	The way that 'we do things' in our organizations and work units, items generally reflect a supportive work culture	**H2**: Care providers who perceive a more positive unit culture report higher research use	My organization effectively balances best practice and productivity
Evaluation ^1^	The process of using data to assess group/team performance and to achieve outcomes in organizations or units	**H3**: Care providers who perceive a larger number of unit feedback mechanisms report higher research use	Our team routinely monitors our performance with respect to the action plans
Social Capital ^1^	The stock of active connections among people. These connections are of three types: bonding, bridging, and linking	**H4**: Care providers who perceive more positive unit social capital activities report higher research use	People in the group share information with others in the group
Formal Interactions ^2^	Formal exchanges that occur between individuals working within an organization (unit) through scheduled activities that can promote the transfer of knowledge	**H6**: Care providers who perceive a larger number of formal unit interactions report higher research use	How often do these activities occur? -Team meetings
Informal Interactions ^2^	Informal exchanges that occur between individuals working within an organization (unit) that can promote the transfer of knowledge	**H5**: Care providers who perceive a larger number of informal unit interactions report higher research use	How often do you interact with people in the following roles or positions? - Someone who *champions *research and its use in practice
Structural/Electronic Resources ^3^	The structural and electronic elements of an organization (unit) that facilitate the ability to assess and use knowledge	**H7**: Care providers who perceive a larger number of unit structural and electronic resources report higher research use	How often do you use/attend the following? - A Library
Organizational Slack	The cushion of actual or potential resources which allows an organization (unit) to adapt successfully to internal pressures for adjustments or to external pressures for changes		
• Human Resources (staffing)^1^		**H8**: Care providers who perceive sufficient unit staffing levels report higher research use	Enough staff to deliver quality care
• Time ^1^		**H9**: Care providers who perceive having sufficient time on their unit report higher research use	Time to do something extra for patients
• Space ^1^		**H10**: Care providers who perceive having sufficient space on their unit report higher research use	Use of designated space

^1 ^= Scale: 1-strongly disagree; 2-disagree; 3-neither agree or disagree; 4-agree; 5-strongly agree

^2 ^= Scale: 1-never; 2-rarely; 3-ocasionally; 4-frequently; 5-almost always

^3 ^= Scale: 1-never; 2-rarely; 3-ocasionally; 4-frequently; 5-almost always; 6-not accessible

The reduced (56-item) version of the ACT was pretested for feasibility and completion time with pediatric nurses in two hospitals in Alberta (Canada). In the pretest, the 56-item ACT was embedded in a larger survey consisting of 135 items and administered to 249 nurses. The mean time to complete the entire survey was 22 minutes for those who completed it online (n = 209) and 33 minutes for those who completed it using paper (n = 40), resulting in average *item *to completion times of 9.8 seconds for the online administration and 14.7 seconds for the paper administration. Using this average item time, we estimated a mean time to completion for the 56-item ACT of 9.1 minutes (when administered online) and 13.7 minutes (when administered by paper), both significantly less than time to completion for the original (76-item) version. Based on these completion times, we decided to administer the ACT in the larger multi-site study (reported in this paper) in online format only.

### Design, sample, and data collection

Seven pediatric hospitals in six Canadian provinces provided the sampling pool for the administration of the English version of the refined 56-item ACT (henceforth simply the ACT). Five healthcare professional subgroups were eligible to participate: nurses, physicians, allied professionals, educators/clinical specialists, and managers. Inclusion and exclusion criteria for the professional subgroups are summarized in Additional File 2. Data were collected using an on-line survey. Eligible participants were provided with a survey package containing a letter introducing the study, and a business card providing a Uniform Resource Locator (URL) and unique password to access the survey on-line.

Ethical approvals for the study were obtained from the appropriate universities and hospital review boards in the respective Canadian provinces.

### Data analysis

Data analyses (except aggregation statistics) were carried out using the Statistical Package for the Social Sciences for Windows (SPSS, v. 16.0) on data collected from professional nurses (n = 752); aggregation statistics were carried out using the SAS 9.2 statistical program. Data analyses included a missing-values analysis; items with missing values greater than 10% were considered for removal and/or imputation [17]. Descriptive statistics (variance, mean, histograms) were generated for each item and examined for amount of variance and middle range mean scores as well as sufficient endorsement frequency. Items with a very high or low frequency (endorsement frequency) were considered for elimination since answers can be predicted with frequencies greater than 80% accuracy and below 20% [18]. These frequencies would also have no influence on the scale's psychometric properties and may increase burden by making it longer.

#### Validity

Since this was the first major field assessment of the ACT, our assessment was largely exploratory rather than confirmatory in nature. Therefore, to examine the underlying dimensional structure of the ACT, we performed factor analysis using principal component analysis (PCA) with orthogonal (Varimax) rotation rather then other factor-analytic methods such as 'principal axis factoring' or 'common factor analysis'. Missing values, which were limited, were treated as such with no substitution or imputation of estimated values. Factors were identified using the 1.0 eigenvalue cutoff rule and the Scree test. Item retention was based on coefficient values (factor loadings ≥ 0.35). Items that cross loaded (factor coefficients ≥ 0.35) on two or more factors were examined on a case-by-case basis and were either re-conceptualized or eliminated from the scale to achieve a balance of good estimation and avoidance of overcapitalizing on sampling error [19]. The Varimax rotation with Kaiser normalization, as recommended by Kline [20], was used to enhance interpretability of the principal component analysis. Following factor analysis, corrected item-total correlations were reviewed for items within the factors identified; items that correlated with the total score below 0.30 were considered for deletion [21]. Items were also considered for deletion if they: (1) caused a significant increase in scale alpha values if they were deleted (item-total statistics), or (2) were highly correlated (> .70 from item-to-item correlations) with each other [22].

The ACT was developed to measure organizational context and was motivated by a need to build a better understanding of how to design effective interventions that result in better research uptake. Several studies examining the impact of context on research implementation in both the nursing [2,23-26] and organizational behaviour literature [27] support the importance of contextual factors to research use. We assessed construct validity of the ACT by examining associations between each of its factors and instrumental research utilization. Instrumental research utilization was defined to study participants as the use of observable research-based practices when caring for patients and was scored on a 5-point frequency scale from 1 (use less then 10% of the time) to 5 (use almost 100% of the time). This item has been used in several previous studies [28-30]. Items within each ACT concept were averaged (culture, leadership, evaluation, social capital, organizational slack-human resources, organizational slack-time, organizational slack-space) or recoded as existing or not existing and then counted (informal interactions, formal interactions, structural and electronic resources) to calculate one derived score for each factor.

While research utilization and the ACT variables were measured and analyzed at the individual level in the study reported in this paper, individual scores on the ACT can be aggregated to obtain unit scores by calculating group means. Therefore, we also calculated a set of indices to assess each identified factor's performance when aggregated. One-way analysis of variance (ANOVA) was performed for each variable using the unit as the group variable. The source table from the one-way ANOVA was used to calculate the following indices: (1) interclass correlation ICC (1) = (BMS - WMS)/(BMS + [K - 1] WMS), where BMS is the between-group mean square, WMS is the within-group mean square, and K is the number of subjects per group. The average K for unequal group size was calculated as K = (1/[N - 1]) (ΣK - [ΣK^2^/ΣK]); (2) interclass correlation ICC (2) = (BMS - WMS)/BMS; (3) η^2 ^= SSB/SST, where SSB is the sum of squares between groups and SST is the sum of squares total; and (4) ω^2 ^= (SSB - [N - 1]WMS)/(SST + WMS). For each variable analyzed, there is strong agreement among nurses in each given unit when ICC (1) is greater than 0.1. Aggregated data are considered reliable when the *F *statistic from the ANOVA table is statistically significant (*p *< 0.05) and/or ICC (2) is greater than 0.60 [31]. An indicator of effect size is η^2^, the proportion of variance in the individual factor accounted for by group membership [32]. Omega squared (ω^2^) is a measure of the relative strength of the aggregated variable at the group level [33]. Both η^2 ^and ω^2^are measures of validity of the aggregated data at the patient care unit level.

#### Reliability

Reliability of the factors within the ACT instrument was examined using Cronbach's alpha (α). Factors below the acceptable standard (0.70) for scales intended to compare groups were considered for revision [21,34].

## Results

### Sample characteristics

The overall response rate for professional nurses completing the ACT in English was 43.5% (n = 764). Twelve cases were deleted (7 cases for having completed less than 90% of the survey, and 5 cases for not meeting eligibility criteria) leaving an analytic sample of 752. A summary of the demographic data pertaining to the final sample completing the ACT in English is presented in Table 2.

**Table 2:** Characteristics of Study Sample (n = 752)

Demographic Characteristic		n (%)
Gender [n, (%)]	Male	32 (4.3)
	Female	720 (95.7)
	*Missing Values*	0
		
Age [n, (%)]	20-24 years	70 (9.3)
	25-29 years	221 (29.4)
	30-34 years	132 (17.6)
	35-39 years	73 (9.7)
	40-44 years	72 (9.6)
	45-49 years	75 (10.0)
	50-54 years	63 (8.4)
	55-59 years	39 (5.2)
	60-64 years	6 (0.8)
	65-70 years	0
	*Missing Values*	1 (0.1)
		
Highest Education Level [n, %]	Diploma/Certificate	250 (33.2)
	Bachelors Degree	488 (64.9)
	Masters Degree	13 (1.7)
	Other	0
	*Missing Values*	1 (0.1)
		
Number of Years Worked in Current Position [Mean (SD)]		8.4 (8.3)
		
Number of Years Worked on Unit [Mean (SD)]		7.7 (7.4)
		
Overall instrumental research utilization score (mean, SD)		3.47 (1.213)

### Missing values and descriptive statistics

We used listwise deletion to deal with missing data. No individual ACT items were missed by greater than 10% of respondents. Item distribution of the 56 ACT items showed acceptable variance and middle range mean scores.

### Validity - Internal structure

#### Factor analysis

Listwise deletion resulted in a final sample of 704 participants for the PCA. The PCA indicated a 13-factor solution accounting for 59.26% of the variance in 'organizational context'. The range of loadings for each factor, along with the means (and standard deviations) for each factor are shown in Table 3.

**Table 3:** ACT Domains: Psychometric Validity and Reliability

					Factor Analysis^1 ^(n = 704)			Cronbach Alpha^2^
Survey Concept	No. Items	No. Completed Responses	Mean Response	Standard Deviation	Factor ranks	Factor Loadings (Range)	Eigenvalue	
**Leadership**	6	750	3.53	0.81	2	0.753 - 0.842	3.825	0.91
**Culture**	6	746	3.83	0.53	7	0.389 - 0.701	1.725	0.72
**Evaluation**	6	747	2.98	0.82	1	0.774 - 0.864	9.806	0.91
**Social Capital**	6	742	3.90	0.47	3	0.584 - 0.684	3.412	0.77
Formal Interactions*	4	745	1.84	1.02	10	0.369 - 0.702	1.286	0.60
Informal Interactions*								
*Type 1- Non-direct Care providers*	4	743	1.09	0.95	4	0.550 - 0.768	2.519	0.75
*Type 2- Direct Care providers*	5	747	4.21	0.86	5	0.339 - 0.798	2.460	0.70
Structural and Electronic Resources*								
*Type 1-Formal Resources*	4	745	2.74	1.00	8	0.439 - 0.806	1.441	0.71
*Type 2- Traditional Resources*	3	745	0.72	0.68	9	0.510 - 0.720	1.406	0.60
*Type 3- Electronic Resources*	3	751	1.36	0.86	13	0.602 - 0.710	1.042	0.54
**Organizational Slack**								
*Time*	4	752	2.93	0.55	6	0.606 - 0.717	1.836	0.74
*Space*	3	750	2.94	0.88	11	0.636 - 0.807	1.232	0.63
*Human Resources*	2	750	2.92	1.03	12	0.759 - 0.788	1.195	0.83

^1^Extraction Method: Principal Component Analysis, Rotation Method: Varimax method

^2^Cronbach alpha was computed for each individual factor. For factors using a count method for deriving scores (formal interactions, informal interactions [Types 1 and 2], structural and electronic resources [Types 1, 2, and 3]), the recoded values were used to compute Cronbach alpha

* Dimensions derived based on count method

#### Culture, Leadership, Evaluation, Structural and Electronic Resources

Culture, leadership, evaluation, and resources constitute organizational context according to the PARiHS framework, which guided the development of the ACT. In our 13-factor solution, the first two factors (eigenvalues 9.806 and 3.825) included all items in the evaluation and leadership subscales and accounted for most of the variance at 17.51% and 6.83%, respectively. The culture items constituted the seventh factor, (eigenvalue 1.725), accounting for 3.08% of the variance. Structural and electronic resources were represented in the eighth, ninth, and thirteenth factors (eigenvalues 1.441, 1.406, and 1.042 respectively) and accounted for 2.57%, 2.51%, and 1.86% of the variance respectively in organizational context. Items with the highest factor coefficients were used to name the three 'types' of structural and electronic resources; type 1 (factor 8) represented *formal resources *(e.g., policies and procedures, clinical practice guidelines); type 2 (factor 9) represented *traditional resources *(e.g., textbooks, journals), and type 3 (factor 13) represented *electronic resources *(e.g., reminder systems, computerized decision support). Together, culture, leadership, evaluation, and structural and electronic resources (context according to the PARiHS framework) accounted for 34.36% of the variance in organizational context as measured by the ACT.

#### Social Capital

The third factor (eigenvalue 3.412) represented social capital and accounted for 6.09% of the variance in organizational context as measured by the ACT.

#### Interactions

Informal interactions were represented in the fourth and fifth factors (eigenvalues 2.519 and 2.460) and accounted for 4.50%, and 4.39% of the variance respectively for a total explained variance of 8.89%. Items with the highest factor coefficients were used to name the two 'types' of informal interactions; type 1 (factor 4) represented interactions with non-direct care providers (e.g., interactions with a clinical educator, interactions with a quality improvement specialist) while type 2 (factor 5) represented interactions with direct care providers (e.g., interactions with other nurses, hallway talk). Formal interactions (e.g., team meetings, patient rounds) were represented in the tenth factor (eigenvalue 1.286) and accounted for an additional 2.30% of the variance in organizational context as measured by the ACT.

#### Organizational Slack

The sixth, eleventh, and twelfth factors (eigenvalues 1.836, 1.232, and 1.195 respectively) represented the three sub dimensions of organizational slack - time, space, and human resources (staffing). These sub dimensions accounted for 3.28% (time), 2.20% (space), and 2.13% (human resources) of the variance for a combined variance of 7.61% in organizational context as measured by the ACT.

#### Item-total statistics

Corrected item-total correlations for items within each of the 13 factors, with the exception of one item, (continuing education in the formal interaction factor, item-total correlation = .231) were greater than the predetermined cutoff of .30 indicating items within each factor were related to the overall scale for that factor. Item-total statistics (alpha when item deleted) for each factor also remained stable, providing further internal structure validity evidence for the ACT.

### Construct validity

To assess construct validity of the ACT we examined associations between the 13 ACT factors and levels of the dependent variable (instrumental research utilization). Increases in each of the 13 factors showed a positive bivariate correlation with an increasing trend from lowest level of instrumental research use to the highest (see Table 4). The p-values for both the Pearson's correlation coefficient and the nonparametric Spearman's rank correlation coefficient show a significant bivariate relation between 12 of the 13 ACT factors and instrumental research use at the 5% level; the only exception was the organizational slack-human resources factor.

**Table 4:** Assessment of Construct Validity: Correlation of derived ACT factors by increasing levels of instrumental research utilization (IRU)

		Mean value (or relative ▽ %) of ACT factors by increasing levels of instrumental research utilization						
	Bivariate correlation with IRU*	1	2	3	4	5	Total^†^	P-value for means differences
Leadership	0.090* ^1^/0.098** ^2^	3.37 (-4.5)	3.45 (-2.3)	3.51 (-0.6)	3.51 (-0.6)	3.68 (4.2)	3.53	0.069^3 ^(0.089^4^)
Culture	0.145**/0.148**	3.68 (-3.9)	3.75 (-2.1)	3.80 (-0.8)	3.83 (0.0)	3.96 (3.4)	3.83	0.002 (0.002)**
Evaluation	0.130**/0.145**	2.64 (-11.4)	2.85 (-4.4)	3.06 (2.7)	2.96 (-0.7)	3.15 (5.7)	2.98	0.000 (0.000) **
Social capital	0.120**/0.119**	3.82 (-2.1)	3.83 (-1.8)	3.89 (-0.3)	3.89 (-0.3)	4.02 (3.1)	3.90	0.009 (0.027) **
Formal Interactions	0.154**/0.156**	1.49 (-19.0)	1.65 (-10.3)	1.91 (3.8)	1.79 (-2.7)	2.14 (16.3)	1.84	0.000 (0.000) **
Informal Interactions (*Type 1- Non-Direct Care Providers*)	0.183**/0.208**	0.56 (-48.6)	0.75 (-31.2)	1.24 (13.8)	1.10 (0.9)	1.31 (20.2)	1.09	0.000 (0.000) **
Informal Interactions (*Type 2- Direct Care Providers*)	0.196**/0.220**	3.69 (-12.4)	4.04 (-4.0)	4.22 (0.2)	4.24 (0.7)	4.45 (5.7)	4.21	0.000 (0.000) **
Structural and Electronic Resources (*Type 1-Formal Resources*)	0.242**/0.240**	2.32 (-15.3)	2.37 (-13.5)	2.76 (0.7)	2.77 (1.1)	3.13 (14.2)	2.74	0.000 (0.000) **
Structural and Electronic Resources (*Type 2- Traditional Resources*)	0.129**/0.137**	0.51 (-29.2)	0.69 (-4.2)	0.69 (-4.2)	0.73 (1.4)	0.89 (23.6)	0.72	0.003 (0.006) **
Structural and Electronic Resources (*Type 3- Electronic Resources*)	0.177**/0.186**	1.11 (-18.4)	1.00 (-26.5)	1.42 (4.4)	1.42 (4.4)	1.56 (14.7)	1.36	0.000 (0.000) **
Organizational Slack-*Human resources*	0.023/0.037	2.76 (-5.5)	2.84 (-2.7)	2.94 (0.7)	3.02 (3.4)	2.85 (-2.4)	2.92	0.251 (0.289)
Organizational Slack-*Space*	0.149**/0.158**	2.63 (-10.5)	2.84 (-3.4)	2.91 (-1.0)	2.95 (0.3)	3.17 (7.8)	2.94	0.000 (0.001) **
Organizational Slack-*Time*	0.137**/0.156**	2.68 (-8.8)	2.90 (-1.4)	2.91 (-1.0)	2.97 (1.0)	3.04 (3.4)	2.94	0.000 (0.001) **

* Indicate significance at 0.05 levels.

** Indicate significance at 0.01 levels.

▽ %: Is the % difference with respect to the total sample average^†^

^1 ^Spearman's rank correlation coefficients

^2 ^Pearson's correlation coefficients

^3 ^P-value for ANOVA

^4 ^P-value for Kruskal-Wallis test

We also assessed the relative percent difference in mean score of each of the ACT's 13 factors from the sample average (Table 4). The results showed a positive incremental relationship with increasing levels of instrumental research utilization (i.e., the higher the contextual scores for each factor assessed relative to the sample average, the better the level of research utilization).

### Internal reliability estimations

Table 3 lists the Cronbach alpha coefficients for each of the 13 factors within the ACT. Coefficients ranged from a low of .54 (for structural and electronic resources - type 3 [electronic resources]) to a high of .91 (for leadership and evaluation factors). With the exception of four factors (structural and electronic resources - type 2 [traditional resources] and type 3 [electronic resources], formal interactions and organizational slack-space) all exceeded the acceptable standard (> 0.70) for scales intended to compare groups recommended by Nunnally and Bernstein [21] and Altman and Bland [34].

### Aggregation of the measures to unit level

The ACT is intended to provide responses that can be aggregated to the level of the patient care unit or to higher organizational levels depending on the context of care delivery for the group(s) completing the instrument. Therefore, we also assessed the performance of the ACT factors when aggregated to the unit level. When developing the ACT, items within the various dimensions were constructed to direct respondents' attention to common experiences on a particular patient care unit in order to ensure that the ACT was meaningful at the unit level. For example, lead-in instructions to the various ACT dimensions asked respondents to assume the shared perspective of their patient care unit (e.g., on my unit we....). Designing items in this way brings about less within-group variability and more between-group variability compared to traditional survey items that focus on individual experiences and perceptions [35,36].

To statistically assess our belief that observations on the ACT are correlated within distinct patient care units, we used four commonly examined aggregation statistics: ICC (1), ICC (2), η^2^, and ω^2 ^(see Table 5) [33,37]. The results supported the reliability of aggregating the ACT factors at the patient care unit level:

**Table 5:** Aggregation of Data to Patient Care Unit Level

Dimensions	F	BMS	WMS	ICC(1)	ICC(2)	Eta^2 ^(η^2^)	Omega^2 ^(ω^2^)	PROB
IRU	2.6142	3.6288	1.3881	0.0569	0.6175	0.0918	0.0566	0.0000
Leadership	8.3876	4.3897	0.5234	0.2164	0.8808	0.2388	0.2101	0.0000
Culture	4.2482	1.0491	0.2470	0.1083	0.7646	0.1377	0.1052	0.0000
Evaluation	6.5082	3.6750	0.5647	0.1708	0.8463	0.1964	0.1660	0.0000
Social Capital	3.1726	0.6443	0.2031	0.0751	0.6848	0.1071	0.0733	0.0000
Formal Interactions	6.4236	5.5822	0.8690	0.1686	0.8443	0.1948	0.1643	0.0000
Informal Interactions (*Type 1- Non-Direct Care Providers*)	3.2734	2.7100	0.8279	0.0783	0.6945	0.1100	0.0763	0.0000
Informal Interactions (*Type 2- Direct Care Providers*)	2.7556	1.9098	0.6931	0.0616	0.6371	0.0938	0.0597	0.0000
Structural and Electronic Resources (*Type 1-Formal Resources*)	5.4840	4.7133	0.8595	0.1436	0.8177	0.1712	0.1398	0.0000
Structural and Electronic Resources (*Type 2- Traditional Resources*)	2.1509	0.9533	0.4432	0.0413	0.5351	0.0749	0.0400	0.0007
Structural and Electronic Resources (*Type 3- Electronic Resources*)	5.4422	3.4771	0.6389	0.1424	0.8162	0.1689	0.1377	0.0000
Organizational Slack-*Human Resources*	8.5578	7.1135	0.8312	0.2203	0.8831	0.2424	0.2139	0.0000
Organizational Slack-*Space*	9.9498	5.8113	0.5841	0.2507	0.8995	0.2712	0.2437	0.0000
Organizational Slack-*Time*	3.5132	0.9827	0.2797	0.0859	0.7154	0.1158	0.0828	0.0000

• **ICC(1)**: The range of ICC(1) values (all greater than 0.00) indicate a degree of perceptual agreement among the nurses about the mean values on the ACT factors within each unit. That is, the nurses' perceptions about context within a particular patient care unit were similar.

• **ICC(2)**: All ACT factors showed statistically significant (*p *< .05) *F *statistics and ICC(2) values greater than 0.60 (with the exception of structural and electronic resources type 2 [traditional resources]), that is, were we to draw repeated subsequent samples from the same groups (units) we would obtain similar mean scores.

• **η^2 ^and ω^2^**: However, the relative effect sizes for both η^2 ^and ω^2 ^values were smaller, suggesting that, as scores on the ACT factors were aggregated, our ability to assign the same meaning for the factor at the unit level as we had at the individual level lessened.

## Discussion

### Reliability

Experts generally disagree on the precise benchmarks that should be applied to psychometric measures such as alpha coefficients. In part, these benchmarks depend on the application. For example, lower Cronbach alpha coefficients (.70 - .80) are deemed acceptable for scales intended to compare groups, while for scales used to measure change within individuals, higher Cronbach alpha coefficients (> .90) are desired [21,34]. The internal consistency of the ACT, in terms of the Cronbach's alpha coefficients of its core dimensions, is for the most part, consistent with usual practice for measures intended to be used at the *level of the group*, or in our case, the patient care unit [34,38]. Only 4 of the 13 ACT factors identified in the factor analysis (structural and electronic resources - type 2 [traditional resources], structural and electronic resources - type 3 [electronic resources], formal interactions and organizational slack-space), had alpha coefficients less than this standard. These lower coefficients may be due to these items addressing concepts that are broader, and perhaps more subject to individual interpretation, than the items in the remaining context concepts.

### Validity - Internal Structure

Factor loadings for all 56 items, with the exception of one item (informal teaching sessions, factor loading = .339) in the ACT exceeded the minimum cut-off of 0.35, indicating that items were representative of underlying factors. Some items did not, however, load as expected. The items on how often respondents participate in 'hallway talk' and 'informal teaching sessions' originally part of the dimension of *formal interactions *loaded with *informal interactions*. Further, the item on how often respondents participate in 'continuing education', originally part of the *structural and electronic resources *dimension, loaded with the *formal interaction *dimension. After careful consideration of these findings the team decided that these loadings were actually a more accurate reflection of the ACT dimensions (as defined in Table 1) that they loaded with and thus we relabelled the item groupings to align with the factor analysis findings.

In developing the ACT we originally hypothesized a 10-factor solution (eight contextual dimensions: culture, leadership, evaluation, social capital, informal interactions, formal interactions, structural and electronic resources, and organizational slack (representing three sub-concepts - time, space, human resources)) with items designated for each concept loading onto a single factor. However, two of our contextual concepts turned out to be multidimensional, loading onto more than one factor (thus yielding a 13-factor solution). Informal interactions had two factors and structural and electronic resources had three factors indicating more complexity to the construct of organizational context than we had originally proposed.

### Construct validity

The validation process in this study demonstrated beginning empirical support for the construct validity of the ACT. Statistically significant bivariate relationships were found between all but one of the ACT's 13 factors at varying levels of instrumental research utilization. That is, higher levels of research utilization were aligned with more positive contextual conditions. Further analyses showed that the mean scores for each of the ACT's 13 factors varied consistently with a positive incremental association between them and reported research utilization levels. These findings are consistent with the PARiHS framework's assertions (see Table 1) and provide additional empirical support for the construct validity of the ACT.

### Aggregation of individual level data to the unit level

Our aggregation statistics indicate that the ACT (when used with professional nurses) can reliably be aggregated to obtain a unit-level assessment of organizational context. We ran the same aggregation statistics with the allied healthcare professionals (n = 209) who completed the ACT survey in the study reported in this paper to explore this further. As we had anticipated, the aggregation statistics did not support aggregation of the ACT with the allied professionals at the unit level; ICC(2) was <.60 for all ACT dimensions indicating low reliability of group means when aggregated to the patient care unit level. Given the differences in how work is constructed for nurses and allied professionals this made sense to us. Most nurses perform their work on a single unit, are aligned with that unit and therefore are able to assess and report on common unit practices, beliefs and values causing them to respond similarly on items examining their patient care unit. Allied professionals such as physiotherapists and respiratory therapists, on the other hand, often work across programs (which consist of several units) and therefore should (and do) display greater within unit variability decreasing reliability of their aggregated response. Therefore, at this point in time we only recommend aggregating responses of professional nurses to obtain unit-level scores on the concepts contained within the ACT.

## Limitations

Although the validation results presented in this manuscript are promising, this initial assessment of the ACT was conducted in one country, with one professional group, and with a moderate size sample. Validation of a newly developed instrument such as the ACT is a longitudinal and multi-step process, requiring numerous positive findings, across a variety of applications and settings. Test-retest reliability was not assessed, so the ACT's stability is unknown. Cross-validation studies are needed to confirm the factor structure obtained in this initial field test and to establish the reliability and validity of the scales in other samples and settings. Additional validation studies using larger sample sizes will be undertaken as additional data are available; these will permit us to extend our assessment to include confirmatory factor analyses and hierarchical linear modeling.

Additional and much longer term investigation is also needed to explore whether overall scores for the multidimensional ACT concepts (e.g., structural and electronic resources, informal interactions, and organizational slack) can be derived from the instrument. At present we are using overall derived scores for each factor as supported by the factor analysis reported in this paper.

## Conclusions

The findings from this initial validation of the ACT must be interpreted with caution and are not generalizable beyond the sample of nurses reported in this manuscript. Although the overall pattern of the data was consistent with the structure hypothesized in the development of the ACT, some items loaded onto their respective factors less strongly than others. This may indicate that respondents are conceptualizing the individual items within a particular concept somewhat differently than we anticipated. This was not totally unexpected, as for the ACT to be applicable to healthcare professionals across a variety of settings, its items were designed to address the respective dimensions as generically as possible. Preliminary work also suggests the instrument in its current form produces its best aggregated results at the unit level among professional nurses.

Follow-up studies are in progress in which we are assessing the ACT with nurses, allied healthcare professionals, physicians, educators and specialists, and managers in long-term care (nursing home) settings, as well as with unregulated (healthcare aide) workers in long-term care settings. Validation of the instrument within the home care sector is also planned. Additional information on the ACT is available from the lead author of this paper.

## Abbreviations

Commonly used abbreviations in this manuscript include: (1) (ACT): Alberta Context Tool; and (2) (PCA): Principal Components Analysis.

## Competing interests

The authors declare that they have no competing interests.

## Authors' contributions

CAE, PGN, JMB, and GGC secured the grant that provided funding for the development of the ACT. CAE and PGN led the statistical analytic design presented in the manuscript. JES assisted in performing and in interpreting the statistical analysis, and made substantial contributions to drafting the manuscript. All authors contributed to the development and revision of the manuscript and approved the final version.

## Pre-publication history

The pre-publication history for this paper can be accessed here:

http://www.biomedcentral.com/1472-6963/9/234/prepub

## Supplementary Material

Additional file 1 **Act Refinement**. A summary of the refinement of ACT from the index (76-item) version to the version piloted. Click here for file

Additional file 2 **Inclusion and Exclusion Criteria by Professional Group**. A summary of the inclusion and exclusion criteria for the professional subgroups. Click here for file
