# Supplementary material for: Development and assessment of the Alberta Context Tool
Source: BMC Health Serv Res. 2009 Dec 15;9:234. doi: 10.1186/1472-6963-9-234 (PMC2805628; doi:10.1186/1472-6963-9-234)
Supplement: Additional file 1 — Act Refinement. A summary of the refinement of ACT from the index (76-item) version to the version piloted. [file 1472-6963-9-234-S1.DOC]

**Additional File 1**

# **ACT Refinement (Index to Pilot Version)**

The original 76-item ACT was refined to 56-items after evaluating missing value patterns, descriptive and item total statistics, and an exploratory orthogonal principal components analysis, considered in combination with existing conceptual (non-empirical) literature on ‘organizational context’.

**Missing values**

Throughout our analyses we used listwise deletion to deal with missing data. With respect to the individual items within the ACT, 5 of the 76 had greater than 10% missing data. We removed these items from the ACT instrument. Table 1 identifies these items.

**Descriptive, item-total statistics, and item-to-item correlations**

Item distribution of the original 76 ACT items showed a modest amount of variance and middle range mean scores. Corrected item-total correlations for items within each of the hypothesized dimensions of context were greater than the predetermined cutoff of .40 indicating items within each dimension were related to the overall scale for that dimension. Item-total statistics (alpha coefficients when the item was deleted) for each hypothesized context dimension also remained stable. Thus, no items were removed from the ACT based on corrected item-total correlations or item-total statistics. Two items were removed based on an examination of item-to-item correlations as follows. Access to a librarian and access to a library were correlated at .702; Access to a librarian was removed from the ACT while access to a library was retained (structural and electronic resources dimension). Enough staff to deliver *optimal* (quality) care and enough staff to get the best possible *patient outcomes* were correlated at .841; Enough staff to get the best possible *patient outcomes* was removed from the ACT while enough staff to deliver *optimal* (quality) care was retained.

**Non-empirical literature**

Eighteen items were removed from the ACT based on non-empirical literature on ‘organizational context’. Items were removed from the eight context dimensions as follows: leadership (3 items), culture (3 items), evaluation (1 item), structural and electronic resources (3 items), and information sharing social processes (8 items). Reasons for deleting these items included: measurement of individual/personal characteristics versus a unit-level/contextual concept (culture, information sharing social processes), and possible redundancy with other concepts (leadership, structural and electronic resources)

**Factor analysis**

Overall, on the basis of missing data (- 4 items), item-to-item correlations (-2 items), and non-empirical grounds (-18 items), the ACT was reduced from 76 items to 52 items. We conducted an exploratory orthogonal principal components factor analysis with listwise deletion on the 52-item instrument to determine if additional items could be removed. One additional item was identified for deletion.

An item in the *organizational slack – time* dimension cross-loaded onto *Formal Interactions*, on closer examination the item was removed as it was shown to duplicate with another item in the *Formal Interactions* dimension.

**Addition of new items**

In addition to the removal of 25 items as discussed above, we also added 5 additional item to ensure the ACT reflected the key concepts it was intended for. This resulted in the development and addition of 3 new social capital items and 2 new organizational slack- space items. Table 2 summarizes the instrument refinement process, documenting the number of items removed and added from the original 76-item ACT.

Table 1. Variables Removed due to Missing Data

| **Dimension** | **Item** | **Total Sample**  **Size** | **No. of Missing Responses*** | **Missing Percent** |
| --- | --- | --- | --- | --- |
| **Leadership** | Acts on values even if it is at a personal cost | 453 | 88 | 19.4% |
| **Informal Interactions** | Interact with knowledge broker | 453 | 42 | 9.3% |
| **Formal Interactions** | Engage in other activities | 453 | 326 | 72.0% |
| **Structural and Electronic Resources** | Other resources | 453 | 428 | 94.5% |
| **Social Capital** | Individuals who do not participate in group activities will be criticized by others in the group | 453 | 48 | 10.6% |

*includes do not know and missing responses

**Table 2. Summary of ACT Refinement**

| **Dimension** | **Starting number** | **Number of items deleted or transferred due to:** | | | | **New Items Created** | **Final number** |
| --- | --- | --- | --- | --- | --- | --- | --- |
|  |  | **Non-empirical** | **Missing**  **data** | **Correlation & item-totals** | **Initial PCA** |  |  |
| **Leadership** | 10 | -3 | -1 |  |  |  | 6 |
| **Culture** | 8 | -2 |  |  |  |  | 6 |
| **Evaluation** | 7 | -1 |  |  |  |  | 6 |
| **Organizational Slack**  *Staffing*  *Time*  *Space* | 3  5  1 |  |  | -1 | -1^3^ | +2 | 2  4  3 |
| **Social Capital**  *Profession*  *Team* | 6  6 | -6^1^  -2 | -1 |  |  | +3 | 0  6 |
| **Informal Interactions** | 9 | -1 | -1 |  |  |  | 7 |
| **Formal Interactions** | 8 | -2^2^ | -1 |  |  |  | 5 |
| **Structural and Electronic Resources** | 13 | +2^2^  -3 |  | -1 |  |  | 11 |
| Net Number of Items | **76** | **-18** | **-4** | **-2** | **-1** | **+5** | **56** |

^1^The same six questions were asked twice, once with respect to an individual’s profession and a second time with respect to the interdisciplinary team. Due to a desire to capture a unit level perspective of context, the profession scale (6 items) was dropped.

^2^Two items (use of reminder systems and use of computerized decision support) were transferred to Structural and Electronic Resources as they seemed more theoretically aligned with this dimension.

^3^Cross loading with Formal Interactions. A duplicate item of an item in that scale, removed from the instrument. (Item*: Engage in non direct care activities (e.g., rounds, family conferences, journal clubs) during work time.*)
