# Supplementary material for: Development and assessment of the Alberta Context Tool
Source: BMC Health Serv Res. 2009 Dec 15;9:234. doi: 10.1186/1472-6963-9-234 (PMC2805628; doi:10.1186/1472-6963-9-234)
Supplement: Additional file 2 — Inclusion and Exclusion Criteria by Professional Group. A summary of the inclusion and exclusion criteria for the professional subgroups. [file 1472-6963-9-234-S2.DOC]

**Additional File 2.** Inclusion and Exclusion Criteria by Professional Group

| **Professional Group** | **Inclusion Criteria** | **Exclusion Criteria** |
| --- | --- | --- |
| **Physicians** | *In ICU settings:*   - appointment to Department of Paediatrics and admitting privileges to the Hospital - on active practice roster - intensivist - neonatologist - fellow (physician in training to become an intensivist or neonatologist) - primary role is to manage care of patients in the NICU or PICU - have worked on the unit for a minimum of 6 months | - residents or students - physicians not currently engaged in clinical practice - surgeons or anaesthesiologist admitting patients post-operatively to the ICU - paediatricians admitting patients to the ICU |
|  | *In non-ICU setting:*   - appointment to Department of Paediatrics and admitting privileges to the hospital - on active practice roster - general paediatrician or paediatric specialty with academic appointment - fellows - identified by unit managers as a core physician who would be seen on the unit at least weekly - have worked on the unit for a minimum of 6 months | - medical students - residents - physicians not currently engaged in clinical practice |
| **Nurses** | **Graduate nurses & registered nurses**: part-time or full-time on the unit involved in the study *(if >1 unit that is included in the study they will select only one and answer all questions for that particular unit)*  - casual nurses identified by the unit managers   - have worked on the unit for a minimum of 6 months | - student nurse  - clinical trials nurses  - nursing aids/paediatric aids  - Licensed Practical Nurses |
|  | **Licensed practical nurse**: part-time, full-time or casual on the unit involved in the study *(if >1 unit that is included in the study they will select only one and answer all questions for that particular unit)*   - have worked on the unit for a minimum of 6 months | - LPN students  - nursing aides/paediatric aides |
| **Managers** | - unit managers - patient care managers - program managers - have worked on the unit for a minimum of 6 months | - patient care directors  - Senior Executives |
| **Educators & Advanced Practice Nurses** | Must be a *registered nurse* working in one of the following positions:   - Clinical nurse educators - Clinical nurse specialists - Advanced nurse practitioners - Nurse Practitioners - Continuing professional education role - Clinical staff education - Professional practice leaders - Clinical specialists - Quality improvement specialists - Clinical Research Nurses - Patient education (including discharge planning) - have worked on the unit for a minimum of 6 months   - identified by unit managers | -academic staff (primary role as an assistant, associate or full professor)  - clinical instructors whose primary role is supervising nursing students |
| **Allied Health Care Professionals** | **Clinical Pharmacists**   - Have worked on the unit for a minimum of 6 months - identified by unit managers |  |
|  | **Respiratory Therapists**   - Have worked on the unit for a minimum of 6 months - identified by unit managers |  |
|  | **Social Workers**   - Have worked on the unit for a minimum of 6 months - identified by unit managers |  |
|  | **Dieticians**   - Have worked on the unit for a minimum of 6 months - identified by unit managers |  |
|  | **Child Life Specialists**   - Have worked on the unit for a minimum of 6 months - identified by unit managers |  |
|  | **Occupational & Physical Therapists**   - Have worked on the unit for a minimum of 6 months - identified by unit managers |  |
|  | **Speech Language Pathologist**   - Have worked on the unit for a minimum of 6 months - identified by unit managers |  |
